# A mediation analysis of meteorological factors on the association between ambient carbon monoxide and tuberculosis outpatients visits

**DOI:** 10.3389/fpubh.2025.1526325

**Published:** 2025-02-05

**Authors:** Tianfeng He, Xujun Qian, Jing Huang, Guoxing Li, Xinbiao Guo

**Affiliations:** ^1^Department of Occupational and Environmental Health Sciences, Peking University School of Public Health, Beijing, China; ^2^Ningbo Municipal Center for Disease Control and Prevention, Ningbo, China; ^3^The First Affiliated Hospital of Ningbo University, Ningbo, China

**Keywords:** tuberculosis, carbon monoxide, short-term exposure, mediation analysis, health

## Abstract

**Background:**

Ambient carbon monoxide (CO) exposure has been identified as an emerging environmental risk factor contributing to the progression of pulmonary tuberculosis (PTB). However, the epidemiological evidence remains inconsistent. This study aims to investigate the short-term association between low-level CO exposure and PTB outpatient visits in a developing region.

**Methods:**

We conducted a time-series study utilizing a distributed lag non-linear model (DLNM) combined with mediating effect analysis, based on daily CO and PTB cases from 2011 to 2020 in Ningbo, China.

**Results:**

Among all patients with PTB, a 0.1 mg/m^3^ increase in CO concentration was associated with an increased risk of PTB outpatient visits in the single-pollutant model, particularly at lag days 2–6. The maximum relative risk (*RR*) was 1.091 (95%CI, 1.020–1.168, lag 0–2 days). Similarly, the maximum cumulative lag effect of CO exposure was 1.781 (*RR* = 1.781, 95%CI: 1.157–2.742, lag 0–15 days). Subgroup analysis revealed a significant effect of CO exposure in males (*RR* = 1.090, 95%CI: 1.009–1.777, lag 0–3 days), females (*RR* = 1.101, 95%CI: 1.014–1.195, lag 0–3 days), younger individuals (*RR* = 1.097, 95%CI: 1.022–1.178, lag 0–2 days), and during the warm season (*RR* = 1.012, 95%CI: 1.002–1.022, lag 0–4 days). Mediation analysis indicated that temperature had an indirect mediating effect on association between CO and PTB (−0.0065, 95%CI: −0.0130 to −0.0004), while air pressure, visibility, and humidity showed no significant mediating effects.

**Conclusion:**

Our findings indicate that ambient CO exposure, even at low levels, has a short-term impact on PTB in developing regions. Temperature plays a partial mediating role in this relationship. Consequently, it is critical to enhance environmental monitoring and early warning systems to effectively address the prevalence of PTB and the delays in health-seeking behavior.

## Graphical abstract

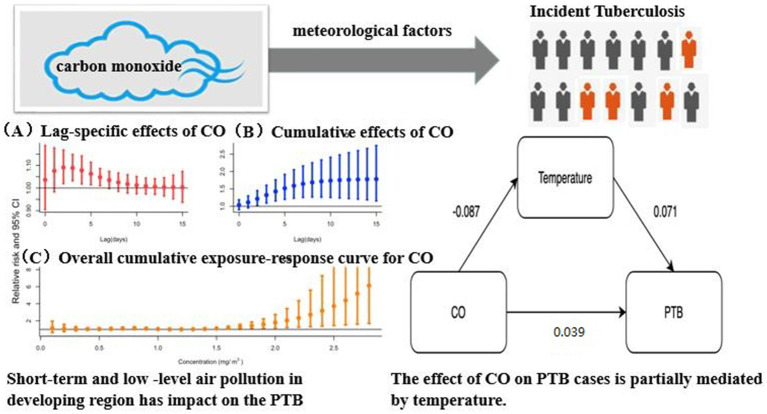

## Highlights

- Short-term and low-level air pollution in developing region has impact on the PTB.
- An increase in CO concentration is related to an increased risk of PTB at lag day 2–6.
- The effect of CO on PTB cases is partially mediated by temperature.
- Climate factors perform mediating role on air pollutants.

## Background

Tuberculosis (TB) is a chronic respiratory infectious disease that significantly impacts human health. In 2021, the global incidence of TB was reported at 133 per 100,000 individuals, accompanied by a mortality rate of 17 per 100,000. Approximately 10.6 million new TB cases and 1.3 million deaths related to TB occurred worldwide (1). Between 2015 and 2021, there was a global decline in TB incidence of approximately 10%, achieving only half of the first milestone set forth by the World Health Organization (WHO) End TB Strategy. China faces a significant burden of TB accounting for approximately 7.36% of new cases globally each year (1). In recent years, the incidence of TB in Ningbo has shown a continuous decline, reaching a rate of 32 per 100,000 in 2021. However, this rate is still far from the WHO’s goal of achieving TB-free status by the year 2035 (2). Consequently, it is imperative to control risk factors such as unhealthy lifestyles, comorbidities (e.g., diabetes and HIV), and environmental exposures to effectively reduce the risk of TB infection (3).

Ambient air pollution has recently been identified as a significant environmental risk factor, contributing to a considerable disease burden by impairing the immune system and increasing oxidative stress and inflammation (4). While most studies have focused on the long-term association between air pollution and TB risks (5), our study investigates the exposure–response relationship and mediating effects of short-term CO exposure on the incidence of PTB. Although some recent studies have explored short-term CO exposure, their findings have been inconsistent, and few have focused on low-pollution regions in developing country. For example, a nested case–control study in Northern California, a developed region, showed that CO exposure was associated with an increased risk of pulmonary TB (6). A study conducted in Shanghai, another high-incidence area in China, reported a relative risk (RR) of 1.031 (95% CI: 1.005–1.057) for a 100 μg/m^3^ increase in CO at an 8-day lag, with similar results observed among male participants, younger age groups, and during the warm-season (3). However, given that Shanghai is characterized by heavy pollution, its findings may be less relevant to our study’s focus on low-pollution regions. An interesting study in Urumqi revealed that PTB incidence increased following 3 months of CO exposure (7). However, two other short-term time-series studies found no significant association between CO and PTB occurrence (8, 9).

Meteorological factors have also been shown to influence the incidence of PTB, including temperature, relative humidity, wind speed, and sunshine duration (10–12). For example, a study in Brazil revealed that PTB incidence was significant correlated with climatic variables such as ultraviolet radiation exceeding 17 MJ/m^2^, relative humidity ranging from 31.0 to 69.0%, and temperatures between 20 and 23°C (10). A Bayesian spatio-temporal study in mainland China identified positive associations between PTB incidence and rainfall, maximum wind speed, and sunshine duration (11). Some studies have also explored the delayed effect of climate factors on PTB, such as Xiao et al.’s (12) finding that average temperature and minimum relative humidity were inversely related to PTB incidence at lag period of 2 and 3–4 months, respectively. A study conducted by Xu et al. (13) based on 22 years of continuous surveillance data from Hong Kong, identified significant associations between certain magnitudes of meteorological factors and increased risks of PTB notifications. Notably, certain meteorological factor, including temperature and humidity, were highlighted in this context (13).

However, few studies have examined the mediating effects of climate factors on the relationship between air pollutants and the incidence of PTB. The aim of this study is to examine the association between CO exposure and incident PTB through mediation analysis. We focus on the association between daily CO exposure levels and the risk of PTB in Ningbo from 2011 to 2020, while also examining the impact of climate factors (such as temperature and humidity) as potential mediators. Furthermore, we explore how variables such as gender, age, and seasonality may influence this relationship. Our findings could provide valuable insights for policymakers in designing targeted air quality standards aimed at mitigating PTB prevalence and safeguarding vulnerable populations.

## Methods

### Study area

**Figure 1:**
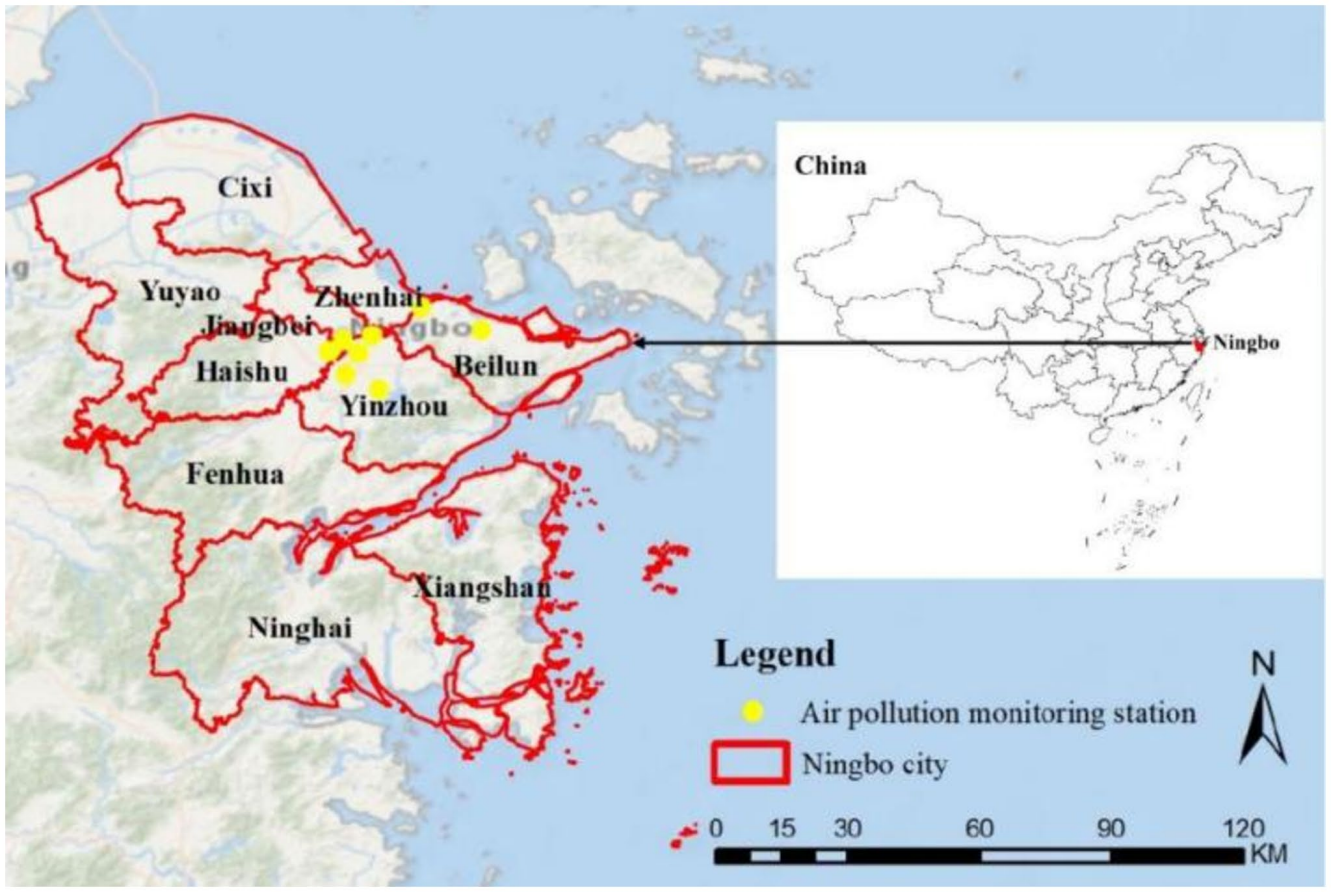
Ningbo city and the locations of air monitoring stations.

Ningbo (center: 121.5°E, 29.9°N) is a coastal city located in Zhejiang Province, China. It covers an area of 9,816 km^2^ and is governed by six districts and four counties, with a permanent population of 9.5 million as of 2021. The daily CO concentrations across the city were estimated by averaging data collected from eight national air monitoring stations located within the core area of Ningbo, as shown in Figure 1.

### Data on PTB

The Center for Disease Control and Prevention of Ningbo manage and report all newly diagnosed PTB cases through an online Tuberculosis Management Information System (TBIMS). For each PTB case, comprehensive information is recorded, including region, age, gender, occupation, date of initial visit, report time, address, disease type and diagnosis unit. For this study, we extracted original case data from the TBIMS for all newly reported PTB cases in Ningbo from January 1, 2011 to July 21, 2020. We included 34,154 newly diagnosed cases, excluding those of patients with relapse or extra-pulmonary tuberculosis. We calculated the daily number of cases and stratified them by age and gender.

### Environmental data collection and mediators

The daily data were obtained from Ningbo China Meteorological Data Network^1^ and the Environmental Monitoring Center, covering the period from January 1, 2011 to July 21, 2020. The average daily concentration of PM_2.5_ (3, 4), NO_2_ (3), CO (3), SO_2_ (3), PM_10_ (4) and 8-h for O_3_ (3) were collected from eight national air monitoring stations. We selected daily meteorological variables, including mean temperature (°C), relative humidity (%), air pressure (Kpa), visibility (m) and wind speed (m/s),as potential mediators in our analysis.

### Statistical analysis

The distributed lag non-linear model (DLNM) is used to examine the lagged effects within the exposure-response relationship (14). In this study, we applied the DLNM to evaluate the association between CO exposure and the risk of PTB outpatient visits. The lag effect in the DLMN captures changes in the outcomes occurring between exposure and subsequent time periods (15). In addition to CO, our preliminary model incorporates four meteorological factors, seasonal variation, and long-term trends (16). To account for long-term trends, we used a “Time” variable (from 1 to 3,490 days). Furthermore, to control for seasonal effects, we incorporated a “7*years” degree of freedom variable (17). A natural cubic spline (ns) function with 3 degrees of freedom was applied to adjust for climate factors, including mean wind speed (WS), temperature (MT), air pressure (AP), and relative humidity (RH) (18). “DOW” and “HOLIDAY” were included as covariates to control for the impact of weekday and holidays, respectively. The single pollutant model is shown as below:


$$Yt\sim \mathrm{quasiPoisson}\;\left(\mu t\right)$$

$$\begin{array}{l} \log\left(\mu t\right)=\alpha +W_{W}^{T}\eta +\mathrm{ns}\left(\mathrm{time},7^{*}\mathrm{year}\right)+\mathrm{ns}\left(MT,df_{1}\right)+\mathrm{ns}\left(WS,df_{2}\right)\\ +\mathrm{ns}\left(RH,df_{3}\right)+{\beta_{1}}^{*}DOW+{\beta_{2}}^{*}\mathrm{HOLIDAY}=\alpha +W_{W}^{T}\eta +\mathrm{COVs} \end{array}$$

Where *t* denotes the observation days. Yt refers to the observed PTB outpatient visits, and μt indicates the anticipated number of visits within t days. $W_{X}^{T}\eta$ refers to the cross-basis function. ns means natural cubic spline function. β_1_ and β_2_ are the regression coefficients for DOW and HOLIDAY. The degrees of freedom for air climate factors are denoted as df1 to df3 (14, 19).

The analysis utilizes the 99.5th quantiles of CO concentration (specifically, 2 mg/m^3^) to assess the impact of CO exposure on PTB outpatient visits. The lag-specific and cumulative risks of PTB outpatient visits per 0.1-unit increase in CO concentration are represented by the Relative Risk (RR) and its corresponding 95% Confidence Interval (CI). Stratified analyses were performed to explore potential heterogeneity in the effects of air pollutants exposure across different age groups, gender, and season. Age was categorized in two groups: ≤65 years and >65 years. Seasons were classified as warm or cold, following the definition in Huang et al. (20). The warm season spans from April to September, while the cold season runs from October to March of the following year. The 95% *CI* of *the RR* estimates is calculated as follows:


$$\left({\hat{Q}}_{1}-{\hat{Q}}_{2}\right)\pm 1.96\sqrt{{\left(S{\hat{E}}_{1}\right)}^{2}+{\left(S{\hat{E}}_{2}\right)}^{2}}$$

Where ${\hat{Q}}_{1}$ and ${\hat{Q}}_{2}$ are the point estimates for the two subgroups, and $S{\hat{E}}_{1}$ and $S{\hat{E}}_{2}$ are the corresponding standard errors, respectively (9). “dlnm” and “splines” packages in R software (version 3.5.2) are used to conduct DNLM.

We further conducted a mediation analysis to estimate the mediating effects of meteorological factors on the association between CO and PTB cases, using 95% confidence intervals. All analysis were conducted using STATA 18.0, and the bootstrap method was applied to assess the impact of meteorological factors and other air pollutants on the relationship between CO and PTB.

## Results

### Descriptive statistics

A total of 34,154 new PTB cases were reported in Ningbo between January 1, 2011 and July 21, 2020, averaging 9.79 cases per day. Among these, 22,936 (67.15%) were male and 11,218 (32.85%) were female, resulting in a male-to-female ratio of 2.04:1. Of the total cases, 29,730 (87.05%) were diagnosed in individuals under 65 years of age, while 5,429 (15.90%) cases were in individuals aged 65 or older at the time of diagnosis. Additionally, 19,120 PTB cases (55.98%) were reported during the warm season, while 15,034 cases (44.02%) occurred during the cold season.

The distribution characteristics of PTB cases, air pollutants, and meteorological factors are shown in Table 1. During the study period, the mean concentration of CO was measured at 0.81 mg/m^3^, with values ranging from 0.02 mg/m^3^ to 2.80 mg/m^3^, which complies with China’s Ambient Air Quality Standards. The average daily incidence of PTB cases was recorded at 9.79, with a range from 0 to 42 cases per day. Supplementary Figure S1 illustrates the time series of monthly average of PTB, PM_2.5_, SO_2_, CO, NO_2_, O_3_, MT, RH, AP, WS in Ningbo from 2011 to 2020. The data reveal distinct seasonal fluctuations as well as long-term trends in the concentrations of air pollutants.

**Table 1:** Descriptive statistics of daily tuberculosis cases, air pollutants concentration and meteorological factors in Ningbo from January 1st 2011 to April 30th 2020.

Variables	Mean ± SD	Minimum	P25	P50	P75	Maximum	IQR
PTB cases	9.79 ± 5.42	0	6	9	13	42	7
Air pollutant							
CO (mg/m^3^)	0.81 ± 0.31	0.02	0.60	0.80	0.99	2.80	0.39
SO_2_ (μg/m^3^)	15.67 ± 12.22	4.00	8.00	12.00	18.00	121.00	10.00
NO_2_ (μg/m^3^)	40.76 ± 18.99	5.00	27.00	38.00	53.00	119.00	26.00
PM_2.5_ (μg/m^3^)	43.50 ± 30.44	4.00	23.00	36.00	55.00	252.00	32.00
O_3_ (μg/m^3^)	90.74 ± 41.03	5.00	61.00	87.00	116.00	255.00	55.00
Meteorological factors							
Mean temperature (°C)	17.66 ± 8.50	−4.30	10.30	18.55	24.60	34.40	14.30
Air pressure (Kpa)	1015.61 ± 8.86	992.30	1008.04	1015.70	1022.51	1039.00	14.47
Relative humidity (%)	75.42 ± 12.38	28.00	67.75	76.00	85.00	100.00	17.25
Wind speed (m/s)	19.42 ± 9.75	0.00	12.00	18.00	25.00	61.00	13.00

PTB, pulmonary tuberculosis; P, percentile; IQR, interquartile range; SD, standard deviation; P25, the 25th percentile; P50, the 50th percentile; P75, the 75th percentile; CO, carbon monoxide; SO_2_, sulfur dioxide; NO_2_, nitrogen dioxide; PM_2.5_, particulate matter with an aerodynamic diameter < 2.5 μm; O_3_, ozone.

Table 2 showed the result of Spearman rank correlation coefficients. CO was negatively correlated with daily PTB cases (*r* = −0.027, *p* < 0.05). Additionally, air pressure and temperature were also found to be negatively correlated (*r* = −0.885, *p* < 0.05).

**Table 2:** Spearman rank correlation coefficients between daily pulmonary tuberculosis cases, air pollutant concentrations and meteorological factors in Ningbo, 2011 to 2020.

Variables	PTB cases	CO	O_3_	SO_2_	NO_2_	PM_2.5_	MT	RH	AP	WS
PTB cases	1.00									
CO	−0.027	1.00								
O_3_	0.103*	−0.182*	1.00							
SO_2_	0.032	0.551*	−0.143*	1.00						
NO_2_	0.006	0.587*	−0.221*	0.646*	1.00					
PM_2.5_	−0.006	0.671*	−0.079*	0.712*	0.731*	1.00				
MT	0.169*	−0.371*	0.388*	−0.378*	−0.497*	−0.412*	1.00			
RH	−0.054*	0.002	−0.346*	−0.380*	−0.115*	−0.236*	0.116*	1.00		
AP	−0.173*	0.309*	−0.326*	0.372*	0.480*	0.378*	−0.885*	−0.214*	1.00	
WS	0.006	−0.189*	0.040*	−0.210*	−0.386*	−0.240*	0.046*	−0.108*	−0.069*	1.00

PTB, pulmonary tuberculosis; CO, carbon monoxide; O_3_, ozone; SO_2_, sulfur dioxide; NO_2_, nitrogen dioxide; PM_2.5_, particulate matter with an aerodynamic diameter < 2.5 μm; MT, mean temperature; RH, relative humidity; AP, air pressure; WS, wind speeds; *P < 0.05 (bilateral).

### The effect of CO exposure on the risk of PTB outpatient visits

**Figure 2:**
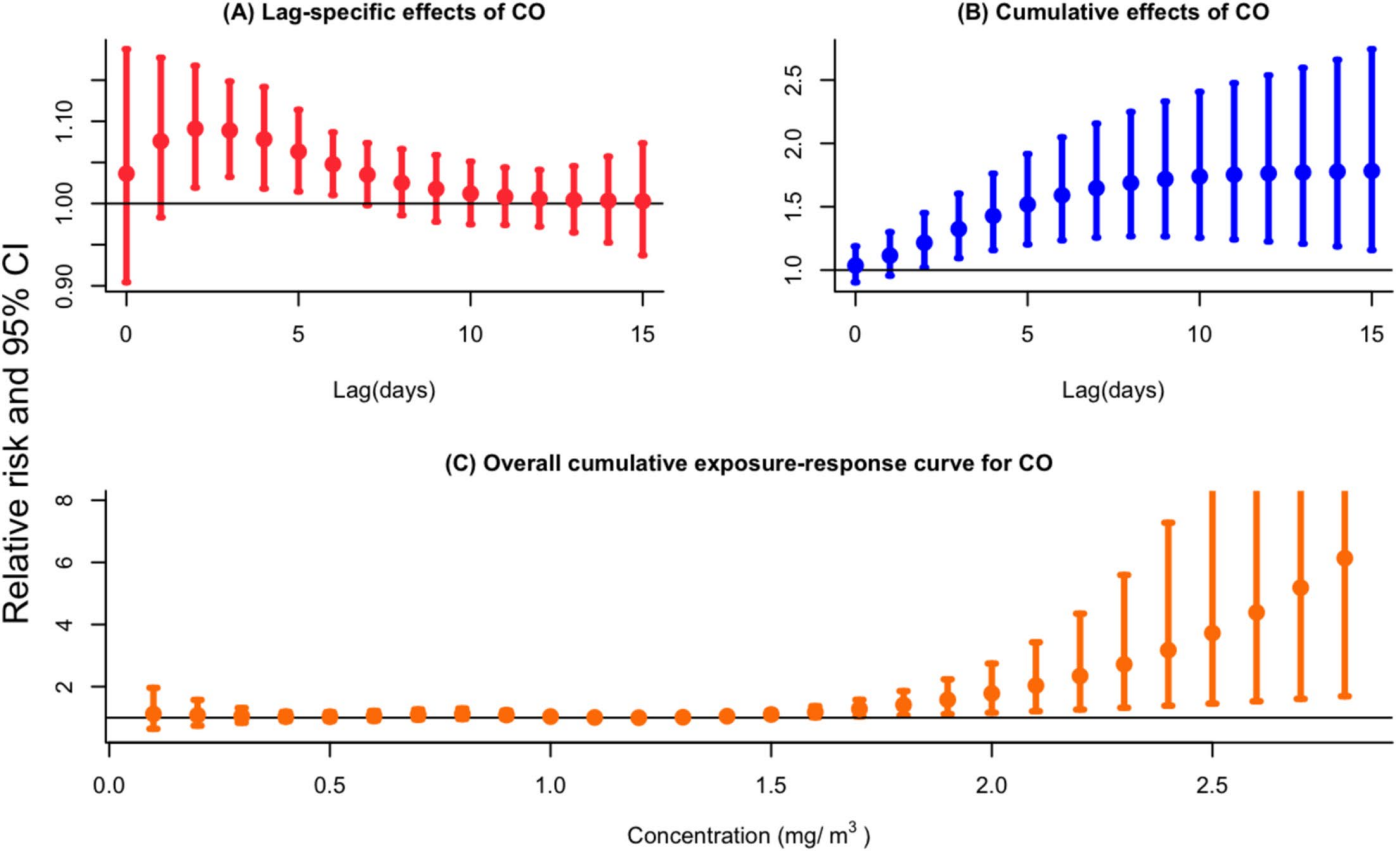
**(A)** Lag-specific relative risks (%) and **(B)** cumulative risks (%) in total outpatient visits for PTB per 0.1 mg/m^3^ increase in daily mean concentrations of CO in the single-pollutant model. **(C)** Overall exposure-response relationships between CO (lag 0–15 day) and the risk of PTB outpatient visits.

As shown in Figure 2A, a 0.1 mg/m^3^ increase in CO concentration was significantly associated with an increase in outpatient visits for PTB from lag days 2 to 6, as demonstrated by the single-pollutant model (*RR* = 1.091, 95%CI: 1.020–1.168, lag 2 day). The maximum cumulative lag effect of CO exposure was statistically significant over a period of 0–15 days (*RR* = 1.781, 95%CI: 1.157–2.742, lag 0–15 days) (see Figure 2B). The 15-day cumulative association between a rise of 0.1 mg/m^3^ in CO concentration and the increased risk of PTB outpatient visits is graphically presented in Figure 2C. The *RR* value reaches its minimum when CO concentration is at 1.2 mg/m^3^, however, *RR* values were statistically significant when the CO concentrations were equal to or greater than 1.6 mg/m^3^.

**Figure 3:**
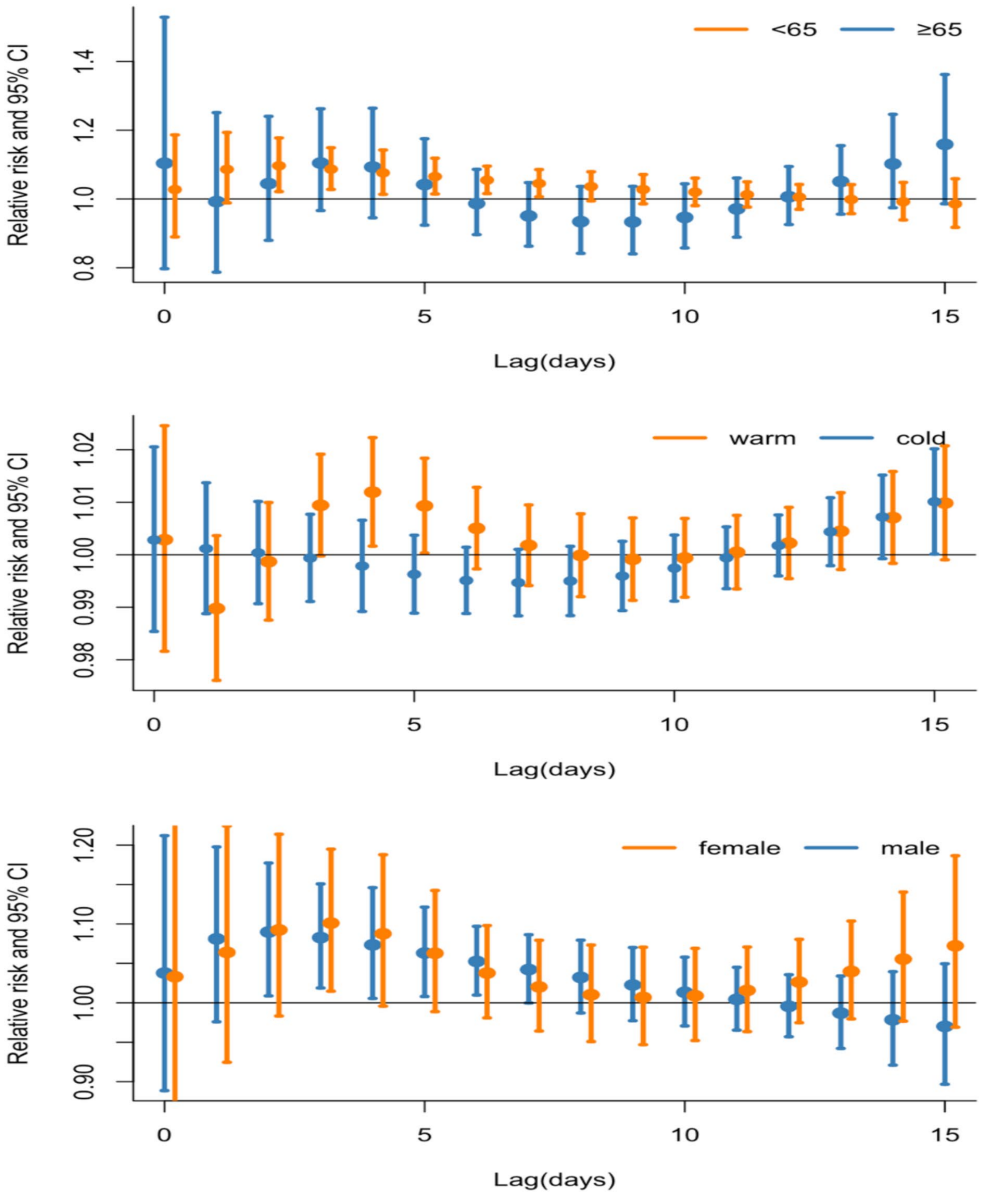
Lag-specific relative risks (95% CI) for outpatient visits to PTB per 0.1 mg/m^3^ increase in daily air pollution concentrations analyzed through single-pollutant models and stratified by age, gender, and season.

In Figure 3, the subgroup analysis indicates that the impact of CO exposure was statistically significant in several groups: among male (*RR* = 1.090, 95%CI: 1.009–1.777, lag 3 day), females (*RR* = 1.101, 95%CI: 1.014–1.195, lag 3 day), younger individuals (*RR* = 1.097, 95%CI: 1.022–1.178, lag 2 day), and during the warm season (*RR* = 1.012, 95%CI: 1.002–1.022, lag 4 day) (Supplementary Table S1).

### Mediation analysis

**Figure 4:**
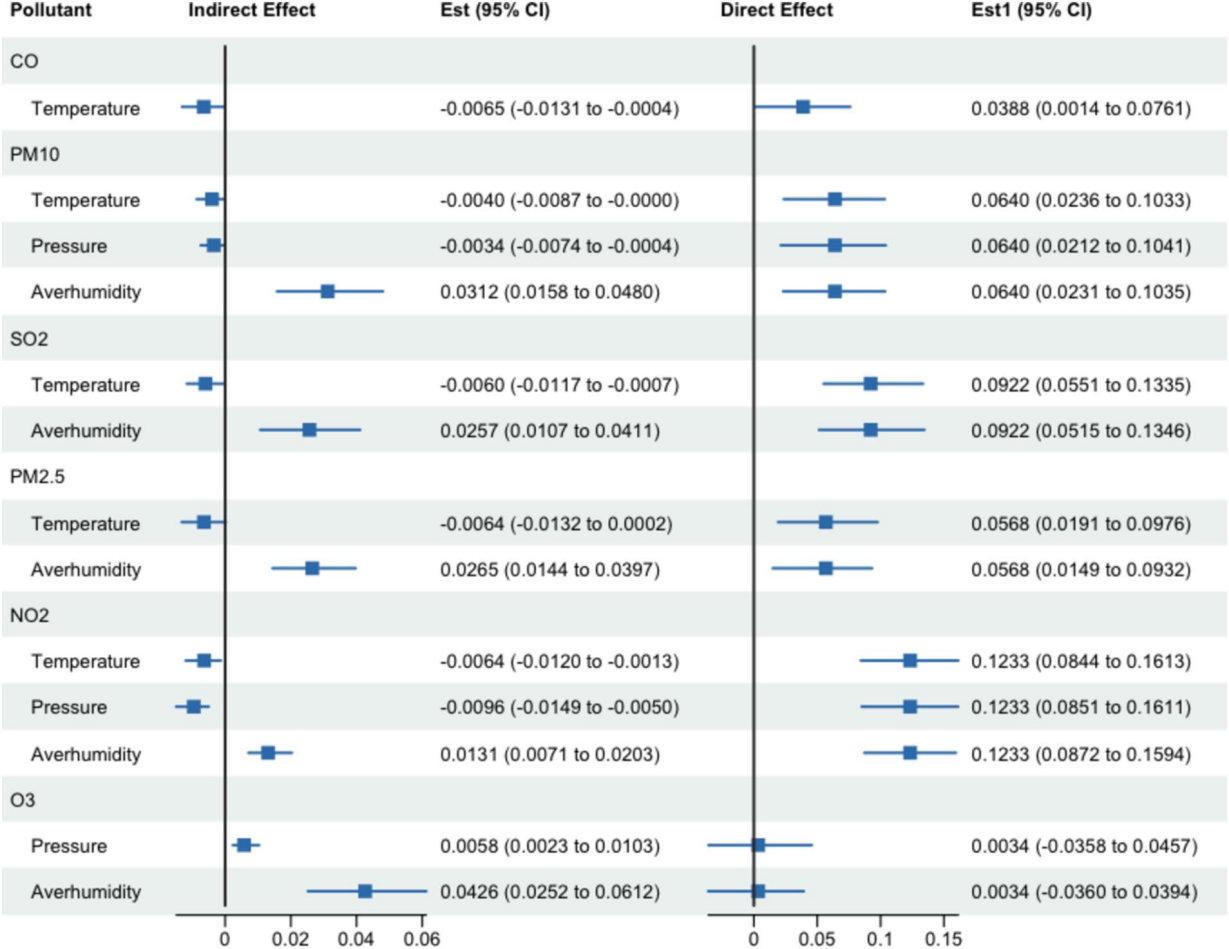
The result of mediating effect of meteorological factors on the association between CO, PM_10_, SO_2_, PM_2.5_, NO_2_, O_3_ exposure and PTB incidence.

This study found that temperature had a mediating effect on the association between CO and PTB −14.35% (95%CI: −0.5, −90.34%) (see Supplementary Figure S2). As other air pollutants did not show a significant association with PTB cases, we further investigated the mediation effects of meteorological factors on the relationship between other air pollutants and PTB cases. Supplementary Table S2 shows that temperature, pressure and humidity all had mediating effects on the association between PM_2.5_, NO_2_ and PTB, respectively. For the association between SO_2_, PM_2.5_ and PTB, temperature and humidity had a mediating effect. For the association between O_3_ and PTB, pressure and humidity had a mediating effect. As the association between SO_2_, PM_2.5_, NO_2_, O_3_, and PTB was influenced by more than one mediator, we further conducted parallel mediation testing to ascertain the cumulative parallel mediation effect for each air pollutant, respectively. The results showed that total parallel mediation effect on each air pollutant ranged from 40.01 to 90.44% (see Figure 4 and Supplementary Table S3).

### Multi-pollutants exposure analysis

To assess the robustness of our findings, we employed a double air pollutants model incorporating CO alongside other ambient air pollutants, including PM_2.5_, SO_2_, O_3_ and NO_2_. Table 3 shows that the *RR* value for CO exhibits minimal variation across the four double-pollutant models, notably, an increase of 0.1 mg/m^3^ in CO concentration is association with an increased risk of PTB outpatient visits from lag period of 2–6 days. Similarly, the *RR* value for all four double pollution exposure reached their peak at a lag time of 2 days.

**Table 3:** Lag-specific relative risks for active PTB cases per 0.1 mg/m^3^ increase in the daily concentrations of CO over lagged 15 days in the multi-pollutant model.

Lag	RR (LCI, UCI)				
	CO	CO and SO_2_	CO and NO_2_	CO and PM_2.5_	CO and O_3_
lag0	1.038(0.905,1.189)	1.025(0.883,1.190)	1.013(0.878,1.168)	1.018(0.861,1.205)	1.041(0.907,1.195)
lag1	1.076(0.983,1.178)	1.074(0.982,1.176)	1.078(0.985,1.180)	1.074(0.981,1.175)	1.077(0.984,1.179)
lag2	**1.091(1.019,1.167)**	**1.090(1.018,1.167)**	**1.094(1.022,1.172)**	**1.092(1.019,1.170)**	**1.091(1.019,1.168)**
lag3	**1.088(1.032,1.148)**	**1.086(1.030,1.146)**	**1.087(1.030,1.146)**	**1.088(1.031,1.148)**	**1.089(1.032,1.149)**
lag4	**1.077(1.017,1.141)**	**1.075(1.015,1.138)**	**1.074(1.014,1.137)**	**1.077(1.016,1.141)**	**1.078(1.018,1.142)**
lag5	**1.062(1.014,1.113)**	**1.060(1.011,1.111)**	**1.059(1.011,1.110)**	**1.062(1.013,1.113)**	**1.062(1.014,1.114)**
lag6	**1.047(1.009,1.086)**	**1.045(1.008,1.084)**	**1.045(1.008,1.084)**	**1.046(1.009,1.086)**	**1.047(1.009,1.085)**
lag7	1.034(0.997,1.073)	1.032(0.995,1.071)	1.033(0.996,1.071)	1.034(0.997,1.073)	1.033(0.996,1.072)
lag8	1.024(0.985,1.065)	1.023(0.983,1.064)	1.024(0.984,1.064)	1.024(0.985,1.065)	1.023(0.984,1.065)
lag9	1.017(0.977,1.058)	1.015(0.975,1.057)	1.016(0.976,1.057)	1.017(0.977,1.058)	1.016(0.976,1.057)
lag10	1.011(0.974,1.050)	1.010(0.972,1.048)	1.010(0.973,1.049)	1.011(0.974,1.050)	1.011(0.973,1.049)
lag11	1.008(0.973,1.043)	1.005(0.971,1.041)	1.006(0.972,1.041)	1.008(0.973,1.043)	1.007(0.973,1.043)
lag12	1.005(0.971,1.040)	1.003(0.969,1.038)	1.003(0.969,1.038)	1.005(0.971,1.040)	1.005(0.971,1.040)
lag13	1.004(0.964,1.045)	1.001(0.961,1.042)	1.000(0.961,1.041)	1.003(0.964,1.045)	1.004(0.964,1.045)
lag14	1.003(0.952,1.056)	0.999(0.948,1.053)	0.998(0.947,1.052)	1.003(0.951,1.056)	1.003(0.952,1.057)
lag15	1.002(0.937,1.073)	0.998(0.932,1.069)	0.996(0.931,1.067)	1.002(0.936,1.073)	1.003(0.937,1.074)

RR, relative risk; UCI, upper confidence interval; LCI, lower confidence interval; PTB, pulmonary tuberculosis; *P* < 0.05 (bilateral); the unit of CO is mg/m^3^.

Bold indicates that the *p* value is less than 0.05, which is statistically significant.

### Sensitivity analysis

Numerous sensitivity analyses were conducted to assess the resilience and validity of our primary findings, exploring various scenarios and conditions: (1) fitting two-pollutant models, (2) changing the length of maximum lag days to 7 and 30 days in the DLNM, (3) using 7 df and 4 df in the splines on time and climate factors, (4) examining the influence of missing data on our DLNM results (i.e., excluding participants with missing meteorological covariates), (5) examining the influence of missing data on our mediation effect results by similarly excluding participants with absent meteorological covariates. While alterations were observed within specific subgroups or pollutants, the primary outcomes remained largely consistent across models involving two pollutants (Supplementary Table S4). This consistency was also evident when modifying maximum lags (Supplementary Figure S3), adjusting degrees of freedom for smoother time and climate factors (Supplementary Figure S4), examining different methods for addressing missing data in relation to DLNM results (Supplementary Figure S5), as well as when examining the influence of missing data on the mediation effect results (Supplementary Tables S4-1–S4-3).

## Discussion

Our findings indicate that the average daily concentration of CO, SO_2,_ NO_2_, PM_2.5_ and O_3_ in Ningbo from lag 0–15 days were 0.81 mg/m^3^, 15.67 μg/m^3^, 40.76 μg/m^3^, 43.50 μg/m^3^ and 90.74 μg/m^3^, respectively. The lag-specific associations between the 97.5th percentiles of CO and the number of PTB cases notified were observed at lag 2–6 days. This association was partially mediating by temperature, with a mediating effect of −14.35% (95% CI: −0.5, −90.34%). The exposure–response curves showed that cumulative risks of PTB cases increased as CO concentrations rose over lags of 2–6 days. This result aligns with a short-term exposure study conducted in Shanghai (3). However, while the precise biological mechanism underlying this association remains unclear, some toxicological studies suggest that CO exposure beyond a certain threshold may impair immune resistance to *Mycobacterium tuberculosis* (*M. tuberculosis*), thereby increasing vulnerability to infection (21). Furthermore, *M. tuberculosis* is the only known organism capable of altering its gene expression in response to varying CO concentrations. When host macrophages produce CO, *M. tuberculosis* activates its CO resistance genes, allowing it to thrive in the presence of CO, unlike other pathogens (22).

Our study found that the 95th percentiles of CO was associated with an increased numbers of PTB cases, consistent with findings from a few studies that reported significant associations between short-term CO exposure and PTB risk (3, 6, 23). For example, a study in Shandong province found a significantly link between short-term outdoor CO exposure and PTB risk (23), and similar results were observed in time-series study in China and case–control studies in the United States (3, 6). However, a study in Wuhan found no significant association between CO and PTB risk in the overall population (8), and similar results were reported from a time-series study in Wuhan based on Kriged Data (9). Despite these conflicting results, our study adds to the growing body of evidence suggesting that short-term exposure to lower CO concentrations may increase the risk for PTB.

We also observed that both male and female patients exposed to low levels of CO had an increased risk of initial PTB; though male patients exhibited a higher risk. These findings are consistent with a population-based case–control study in Northern California (6) and a long-term study in Jinan, China (24). Interestingly, the Jinan study did not identify any association between short-term CO exposure and PTB risk for either gender, highlighting a gap that our study addresses. The heightened sensitivity of males to PTB risk when exposed to lower levels of CO may be attributed to factors such as smoking and occupation exposures. For instance, the prevalence of smoking is significantly higher among men (68.2%) compared to women (11.7%) (25), and it is well-established that smoking increases the risk of PTB (26). Furthermore, males are more likely than females to work in environments such as chemical plants, coal mines, and roads where exposure to air pollutants is higher (27). We also identified a significant positive association between increased CO levels and the risk of PTB during the warm season, despite an inverse mediation effect attributed to temperature. This finding contrasts with studies conducted in Wuhan and Jinan, where air pollution levels, including CO concentrations, were observed to be higher during the cold season than in the warm season (8, 23). However, Ningbo, being a low-level air pollution region, individuals tend to spend more time outdoors during the warm season, this increased outdoor activity may elevate cardiopulmonary load, respiratory rate, and inhalation volume, thereby contributing to greater CO intake (3). Furthermore, younger patients were more likely to be affected by low-level CO exposure.

A key strength of this study is our use of daily time-serious data combined with mediation analysis to explore the association between low-level CO exposure and PTB cases. This approach helps to better understand the impacts of low-levels CO exposure on PTB cases over a 15-day period. Mediation analysis also sheds light on how climate factors may mediate the association between CO exposure and PTB incidence. However, several limitations must be acknowledged. First, our study was limited to a coastal city with relatively low air pollution and specific meteorological characteristics, which may limit the generalizability of our findings to other regions. Given Ningbo’s characteristic, our study may be more representative of other cities in developing regions with low air pollution levels, but larger studies involving more low level air pollution cities are needed. Second, we lacked individual-level data, and the air pollution data used were primarily obtained from fixed monitoring stations, which may not accurately reflect individual exposure. In future studies, wearable devices could be used to collect more accurate air pollution exposure data (28).

## Conclusion

Our findings indicate that low-level exposure to CO is associated with an increased risk of PTB outpatient visits in the short-term. Temperature partially mediates this association. Both male and female, as well as younger individuals, were found to be more susceptible to be effects of low-level CO exposure. Additionally, all patients were more vulnerable to CO exposure during the warm season. These findings highlight the urgent need to establish a comprehensive environmental monitoring and early warning system, with a focus on controlling environmental risk factors, in order to prevent tuberculosis and reduce delays in health-seeking behavior.

## Data Availability

The raw data supporting the conclusions of this article will be made available by the authors, without undue reservation.

## Footnotes

^1^ http://data.cma.cn/
