# Supplementary material for: A mediation analysis of meteorological factors on the association between ambient carbon monoxide and tuberculosis outpatients visits
Source: Front Public Health. 2025 Feb 5;13:1526325. doi: 10.3389/fpubh.2025.1526325 (PMC11841497; doi:10.3389/fpubh.2025.1526325)
Supplement: Supplementary file 1 [file Data_Sheet_1.docx]

**
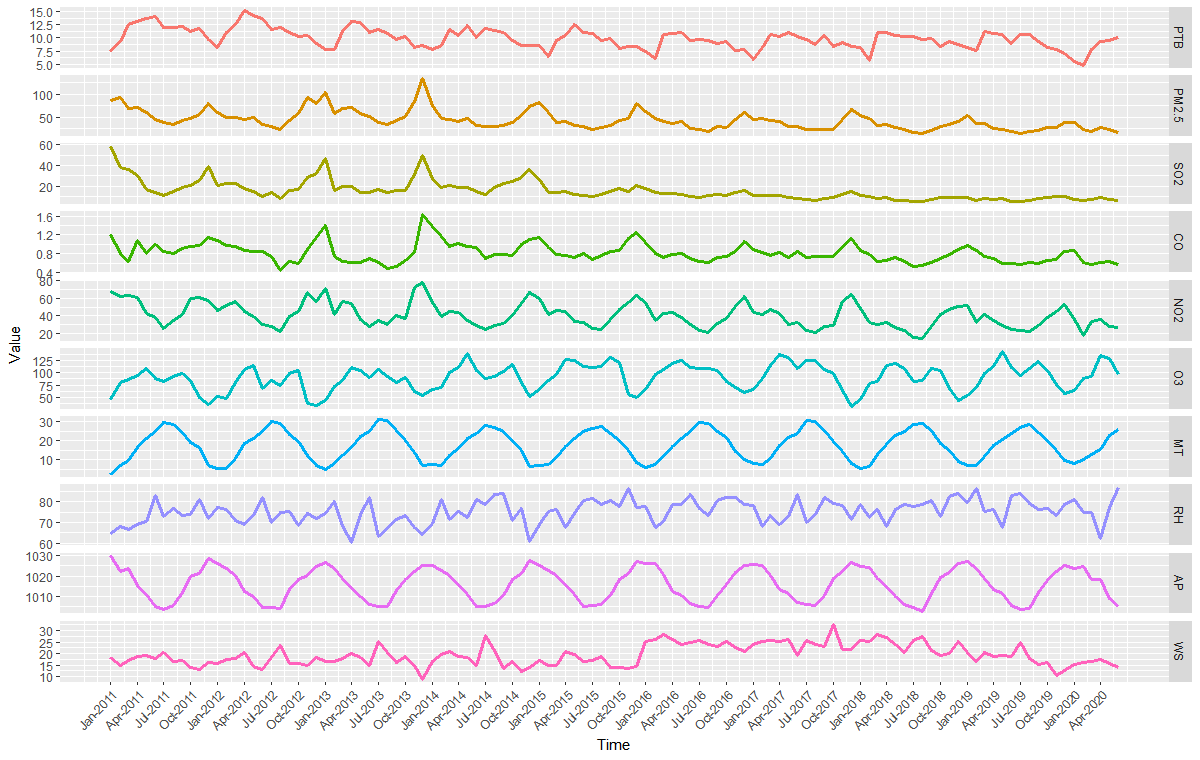
**

**Figure S1** Daily changes of PTB, PM_2.5_, SO_2_, CO, NO_2_, O_3_, MT, RH, AP, WS in Ningbo, China, from January 2011 to July 2020.


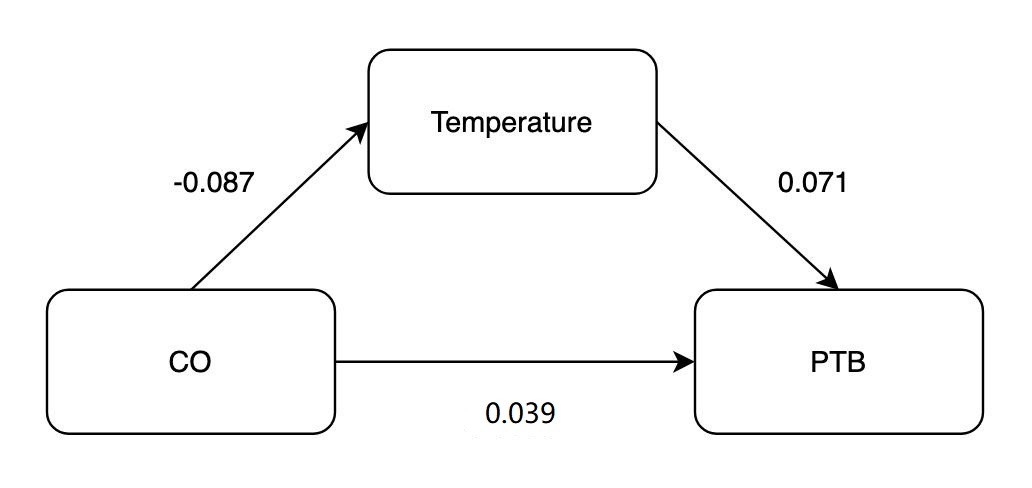


**Figure S2:** Mediation effect analysis of temperature on the association between Co and PTB.


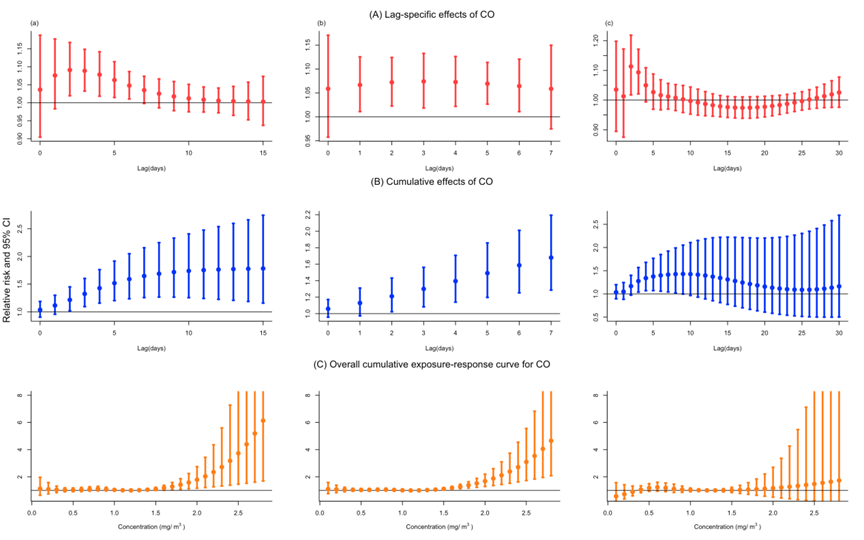


**Figure S3** Sensitivity test DLNM results by change the maximum lag days. (a) cumulative excess risks(%) in initial outpatient visits for TB per 0.1 mg/m^3^ increase in the daily concentrations of CO over lagged 15 days in single- pollutant models. (b) cumulative excess risks(%) in initial outpatient visits for PTB per 0.1 mg/m^3^ increase in the daily concentrations of CO over lagged 7 days in single- pollutant models. (c) cumulative excess risks(%) in initial outpatient visits for PTB per 0.1 mg/m^3^ increase in the daily concentrations of CO over lagged 30 days in single-pollutant models.


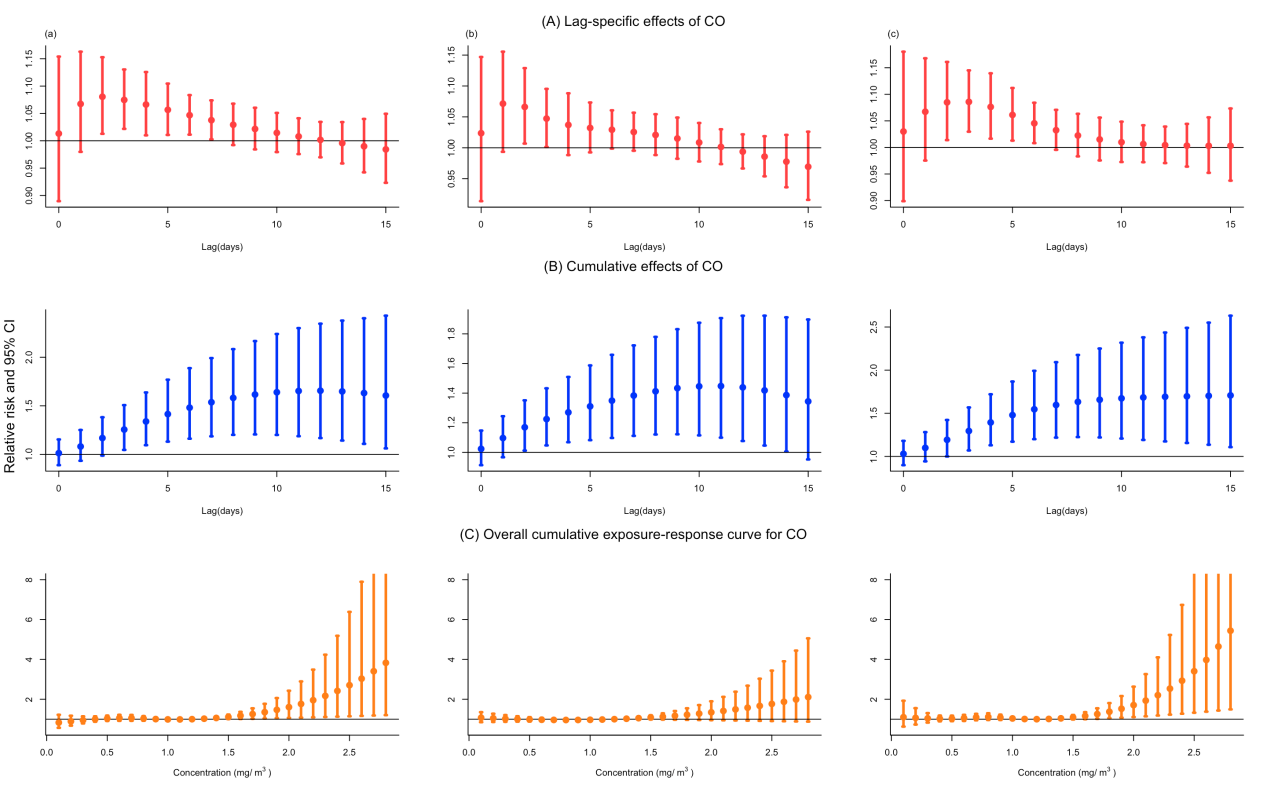


**Figure S4** Sensitivity test DLNM results by change the dfs. (a) cumulative excess risks (%) in initial outpatient visits for PTB per 0.1 mg/m^3^ increase in the daily concentrations of CO under the df 3 in single-pollutant models. (b) cumulative excess risks (%) in initial outpatient visits for PTB per 0.1 mg/m^3^ increase in the daily concentrations of CO under the df 2 in single- pollutant models.(c) cumulative excess risks (%) in initial outpatient visits for PTB per 0.1 mg/m^3^ increase in the daily concentrations of CO under the df 4 in single-pollutant models.


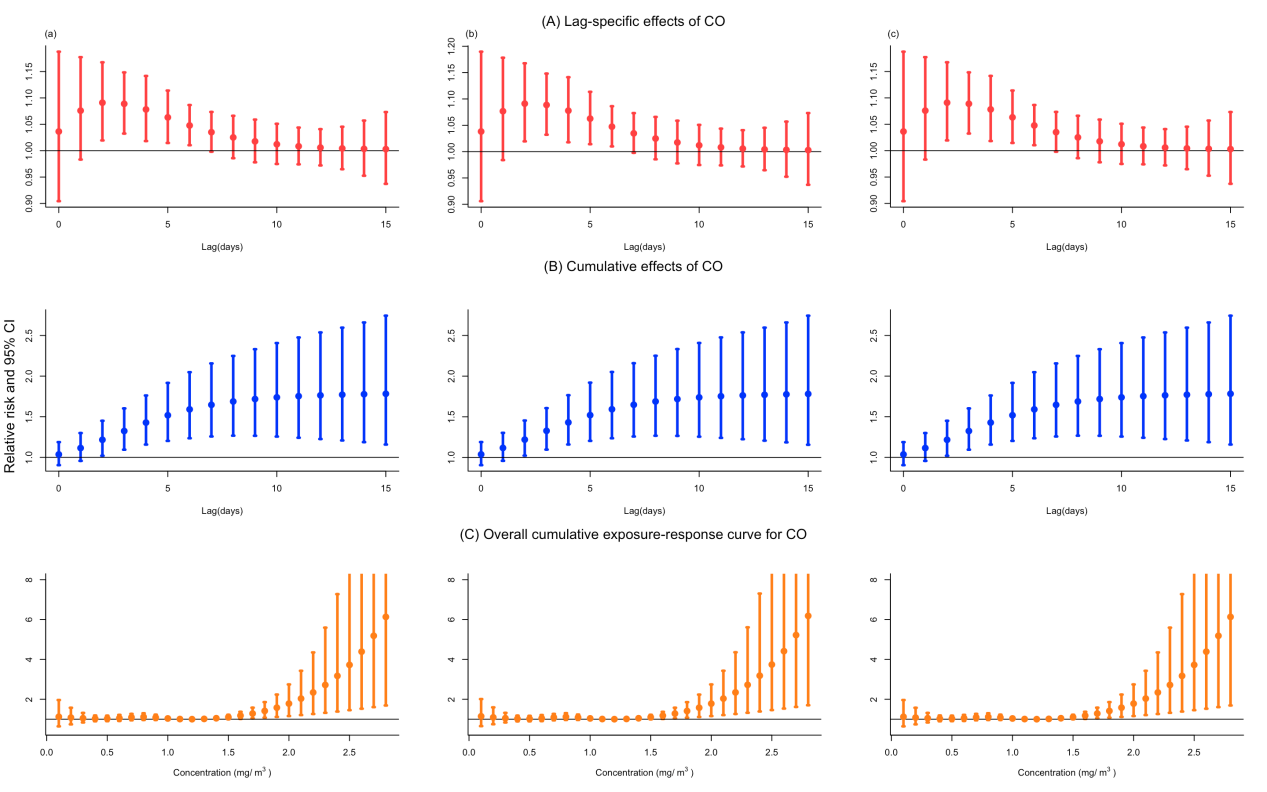


**Figure S5**  Sensitivity test DLNM results by change the methods dealing with missing data. (a) cumulative excess risks (%) in initial outpatient visits for PTB per 0.1 mg/m^3^ increase in the daily concentrations of CO using mean value dealt with missing data of dataset in single- pollutant models. (b) cumulative excess risks (%) in initial outpatient visits for PTB per 0.1 mg/m^3^ increase in the daily concentrations of CO excluding missing data from dataset in single-pollutant models.. (c) cumulative excess risks (%) in initial outpatient visits for PTB per 0.1 mg/m^3^ increase in the daily concentrations of CO using median value dealt with missing data of dataset in single-pollutant models.

**Table S1**  Lag-specific relative risks for active PTB cases per 0.1 mg/m^3^ increase in the daily concentrations of CO over lagged 15 days in the single-pollutant model stratified by age, gender and season

| **Lag** | **RR (LCI, UCI)** | | | | | | |
| --- | --- | --- | --- | --- | --- | --- | --- |
|  | **Total** | **Male** | **Female** | **Elder** | **Young** | **Cold** | **Warm** |
| lag0 | 1.038(0.906,1.190) | 1.040(0.890,1.214) | 1.034(0.837,1.279) | 1.105(0.798,1.530) | 1.029(0.891,1.189) | 1.003(0.985,1.021) | 1.003(0.982,1.025) |
| lag1 | 1.077(0.984,1.178) | 1.082(0.976,1.199) | 1.065(0.926,1.226) | 0.996(0.790,1.255) | 1.087(0.989,1.194) | 1.001(0.989,1.014) | 0.99(0.976,1.004) |
| lag2 | **1.091(1.020,1.168)** | **1.090(1.009,1.177)** | 1.093(0.984,1.214) | 1.045(0.880,1.240) | **1.097(1.022,1.178)** | 1.000(0.991,1.010) | 0.999(0.987,1.010) |
| lag3 | **1.089(1.032,1.148)** | **1.082(1.018,1.151)** | **1.101(1.014,1.195)** | 1.101(0.963,1.259) | **1.087(1.028,1.149)** | 0.999(0.991,1.008) | **1.009(1.000,1.019)** |
| lag4 | **1.078(1.018,1.141)** | **1.073(1.005,1.146)** | 1.087(0.995,1.188) | 1.089(0.942,1.260) | **1.076(1.013,1.143)** | 0.998(0.989,1.007) | **1.012(1.002,1.022)** |
| lag5 | **1.063(1.014,1.114)** | **1.062(1.007,1.121)** | 1.063(0.989,1.143) | 1.04(0.922,1.173) | **1.065(1.014,1.118)** | 0.996(0.989,1.004) | **1.009(1.000,1.019)** |
| lag6 | **1.047(1.010,1.086)** | **1.052(1.009,1.096)** | 1.038(0.981,1.098) | 0.987(0.896,1.086) | **1.054(1.015,1.095)** | 0.995(0.989,1.001) | 1.005(0.997,1.013) |
| lag7 | 1.035(0.998,1.073) | 1.041(0.999,1.086) | 1.021(0.965,1.08) | 0.952(0.864,1.049) | **1.044(1.006,1.085)** | 0.995(0.988,1.001) | 1.002(0.994,1.010) |
| lag8 | 1.025(0.985,1.066) | 1.031(0.986,1.079) | 1.011(0.951,1.074) | 0.936(0.844,1.039) | 1.035(0.994,1.079) | 0.995(0.988,1.002) | 1.000(0.992,1.008) |
| lag9 | 1.017(0.978,1.059) | 1.022(0.977,1.07) | 1.007(0.947,1.071) | 0.937(0.843,1.04) | 1.027(0.985,1.071) | 0.996(0.989,1.003) | 0.999(0.991,1.007) |
| lag10 | 1.012(0.975,1.051) | 1.013(0.970,1.057) | 1.009(0.953,1.070) | 0.950(0.861,1.048) | 1.019(0.980,1.060) | 0.997(0.991,1.004) | 1.000(0.992,1.007) |
| lag11 | 1.008(0.974,1.044) | 1.004(0.965,1.045) | 1.016(0.964,1.071) | 0.975(0.893,1.065) | 1.012(0.976,1.049) | 0.999(0.994,1.005) | 1.001(0.994,1.008) |
| lag12 | 1.006(0.972,1.041) | 0.995(0.957,1.036) | 1.026(0.975,1.08) | 1.011(0.93,1.099) | 1.005(0.969,1.042) | 1.002(0.996,1.008) | 1.002(0.995,1.009) |
| lag13 | 1.004(0.965,1.045) | 0.987(0.942,1.034) | 1.039(0.979,1.103) | 1.055(0.960,1.160) | 0.998(0.957,1.041) | 1.004(0.998,1.011) | 1.004(0.997,1.012) |
| lag14 | 1.003(0.952,1.057) | 0.979(0.921,1.040) | 1.055(0.976,1.140) | 1.106(0.979,1.251) | 0.991(0.938,1.048) | 1.007(0.999,1.015) | 1.007(0.998,1.016) |
| lag15 | 1.003(0.937,1.073) | 0.971(0.897,1.050) | 1.071(0.968,1.185) | 1.163(0.990,1.367) | 0.985(0.917,1.058) | 1.010(1.000,1.020) | 1.010(0.999,1.020) |

Notes: RR, relative risk; UCI, upper confidence interval; LCI, lower confidence interval; PTB,pulmonarytuberculosis;, *P*<0.05;the unit of CO is mg/m^3^.

**Table S2** The mediating effect of meteorological factors on the association between CO, SO_2_, PM_2.5_, NO_2_, O_3_ and PTB.

| **Pollutant** | **Indirect effect est.** | **CI** | **Direct effect est.** | **CI** |
| --- | --- | --- | --- | --- |
| CO |  |  |  |  |
| Temperature | -0.0065 | (-0.0131, -0.0004) | 0.0388 | (0.0014, 0.0761) |
| SO_2_ |  |  |  |  |
| Temperature | -0.0060 | (-0.0117, -0.0007) | 0.0922 | (0.0551, 0.1335) |
| Humidity | 0.0257 | (0.0107, 0.0411) | 0.0922 | (0.0515, 0.1346) |
| PM_2.5_ |  |  |  |  |
| Temperature | -0.0064 | (-0.0132, 0.0002) | 0.0568 | (0.0191, 0.0976) |
| Humidity | 0.0265 | (0.0144, 0.0397) | 0.0568 | (0.0149, 0.0932) |
| NO_2_ |  |  |  |  |
| Temperature | -0.0064 | (-0.0120, -0.0013) | 0.1233 | (0.0844, 0.1613) |
| Pressure | -0.0096 | (-0.0149, -0.0050) | 0.1233 | (0.0851, 0.1611) |
| Humidity | 0.0131 | (0.0071, 0.0203) | 0.1233 | (0.0872, 0.1594) |
| O_3_ |  |  |  |  |
| Pressure | 0.0058 | (0.0023, 0.0103) | 0.0034 | (-0.0358, 0.0457) |
| Humidity | 0.0426 | (0.0252, 0.0612) | 0.0034 | (-0.0360, 0.0394) |

Notes: CI, confidence interval; CO, carbon monoxide; SO_2_, sulfur dioxide; PM_2.5_, particulate matter with an aerodynamic diameter < 2.5µm; NO_2_, nitrogen dioxide; O_3_, ozone; PTB,pulmonarytuberculosis; P<0.05;t he unit of CO is mg/m^3^.

**Table S3** The parallel mediating effect of of meteorological factors on the association between CO, PM_10_, SO_2_, PM_2.5_, NO_2_, O_3_ and PTB.

| **Pollutants** | **Indirect effect** | | **Direct effect** | | **Total effect** | **Total Proportion Mediated** | **Total Indirect effect/ Total direct effect** |
| --- | --- | --- | --- | --- | --- | --- | --- |
|  | EF | 95%CI | EF | 95%CI |  | (%) | (%) |
| SO_2_ | -0.063 | (-0.084 , -0.043) | 0.095 | (0.057 , 0.133) | 0.159 | 40.01% | 66.71% |
| PM_2.5_ | -0.069 | (-0.086 , -0.051) | 0.063 | (0.026 , 0.100) | 0.132 | 52.14% | 108.93% |
| NO_2_ | -0.117 | (-0.137 , -0.096) | 0.123 | (0.086 , 0.161) | 0.240 | 48.65% | 94.73% |
| O_3_ | 0.093 | (0.072 , 0.115) | 0.010 | (-0.029 , 0.048) | 0.103 | 90.44% | 94.63% |

Notes: PTB, pulmonary tuberculosis; EF, effect; SO_2_, sulfur dioxide;PM_2.5_, particulate matter with an aerodynamic diameter < 2.5µm;NO_2_, nitrogen dioxide; O_3_, ozone;.

**Table S4-1** Mediation analysis result by using mean value to deal with missing data among dataset.

| **Mediating factors** | **Temperature** | | **Pressure** | | **Humidity** | |
| --- | --- | --- | --- | --- | --- | --- |
|  | Indirect effect | 95% CI | Indirect effect | 95% CI | Indirect effect | 95% CI |
| CO | -0.0062 | (-0.0133, 0.0004) | 0.0019 | (-0.0004, 0.0046) | 0.0029 | (-0.0011, 0.0069) |
| SO_2_ | -0.0062 | (-0.0114, -0.0009) | -0.0007 | (-0.0033, 0.0018) | 0.0258 | (0.0105, 0.0409) |
| PM_2.5_ | -0.0065 | (-0.0127, -0.0001) | 0.0005 | (-0.0022, 0.0033) | 0.0265 | (0.0147, 0.0402) |
| NO_2_ | -0.0065 | (-0.0118, -0.0014) | -0.0095 | (-0.0146, -0.0049) | 0.0132 | (0.0073, 0.0202) |
| O_3_ | 0.0055 | (-0.0016, 0.0126) | 0.0057 | (0.0023, 0.0100) | 0.0428 | (0.0256, 0.0605) |

Notes: CI, confidence interval; CO, carbon monoxide; SO_2_, sulfur dioxide; PM_2.5_, particulate matter with an aerodynamic diameter < 2.5µm; NO_2_, nitrogen dioxide; O_3_, ozone;.

**Table S4-2** Mediation analysis result by using median value to deal with missing data among dataset.

| Mediating factors | Temperature | | Pressure | | Humidity | |
| --- | --- | --- | --- | --- | --- | --- |
|  | Indirect effect | 95% CI | Indirect effect | 95% CI | Indirect effect | 95% CI |
| CO | 0.0023 | (0.0005, 0.0049) | 0.0019 | (-0.0002, 0.0045) | 0.0029 | (-0.0005, 0.0071) |
| SO_2_ | -0.0062 | (-0.0117, -0.0005) | -0.0006 | (-0.0035, 0.0018) | 0.0258 | (0.0101, 0.0436) |
| PM_2.5_ | -0.0065 | (-0.0128, -0.0008) | 0.0005 | (-0.0022, 0.0033) | 0.0265 | (0.0141, 0.0393) |
| NO_2_ | -0.0065 | (-0.0127, -0.0012) | -0.0095 | (-0.0149, -0.0049) | 0.0132 | (0.0070, 0.0197) |
| O_3_ | 0.0055 | (-0.0014, 0.0122) | 0.0057 | (0.0025, 0.0102) | 0.0427 | (0.0260, 0.0597) |

Notes: CI, confidence interval; CO, carbon monoxide; SO_2_, sulfur dioxide; PM_2.5_, particulate matter with an aerodynamic diameter < 2.5µm; NO_2_, nitrogen dioxide; O_3_, ozone;.

**Table S4-3** Mediation analysis result by using dataset without missing data

| Mediating factors | Temperature | | Pressure | | Humidity | |
| --- | --- | --- | --- | --- | --- | --- |
|  | Indirect effect | 95% CI | Indirect effect | 95% CI | Indirect effect | 95% CI |
| CO | -0.0065 | (-0.0129, -0.0003) | 0.0019 | (-0.0003, 0.0046) | 0.0028 | (-0.0007, 0.0072) |
| SO_2_ | -0.0060 | (-0.0119, -0.0006) | -0.0007 | (-0.0036, 0.0020) | 0.0257 | (0.0099, 0.04056) |
| PM_2.5_ | -0.0065 | (-0.0128, -0.0001) | 0.0005 | (-0.0023, 0.0030) | 0.0265 | (0.0155, 0.0389) |
| NO_2_ | -0.0064 | (-0.0121, -0.0009) | -0.0096 | (-0.0151, -0.0049) | 0.0131 | (0.0067, 0.0196) |
| O_3_ | 0.0054 | (-0.0012, 0.0127) | 0.0058 | (0.0023, 0.0102) | 0.0426 | (0.0265, 0.0602) |

Notes: CI, confidence interval; CO, carbon monoxide; SO_2_, sulfur dioxide; PM_2.5_, particulate matter with an aerodynamic diameter < 2.5µm; NO_2_, nitrogen dioxide; O_3_, ozone;.
